# PprA Protein Inhibits DNA Strand Exchange and ATP Hydrolysis of *Deinococcus* RecA and Regulates the Recombination in Gamma-Irradiated Cells

**DOI:** 10.3389/fcell.2021.636178

**Published:** 2021-04-20

**Authors:** Yogendra Singh Rajpurohit, Dhirendra Kumar Sharma, Hari S. Misra

**Affiliations:** ^1^Molecular Biology Division, Bhabha Atomic Research Centre, Mumbai, India; ^2^Homi Bhabha National Institute (DAE- Deemed University), Mumbai, India

**Keywords:** PprA, DrRecA, *Deinococcus radiodurans*, DNA repair, ATPase

## Abstract

DrRecA and PprA proteins function are crucial for the extraordinary resistance to γ-radiation and DNA strand break repair in *Deinococcus radiodurans*. DrRecA mediated homologous recombination help in DNA strand break repair and cell survival, while the PprA protein confers radio-resistance *via* its roles in DNA repair, genome maintenance, and cell division. Genetically *recA* and *pprA* genes interact and constitute an epistatic group however, the mechanism underlying their functional interaction is not clear. Here, we showed the physical and functional interaction of DrRecA and PprA protein both *in solution* and inside the cells. The absence of the *pprA* gene increases the recombination frequency in gamma-irradiated *D. radiodurans* cells and genomic instability in cells growing under normal conditions. PprA negatively regulates the DrRecA functions by inhibiting DrRecA mediated DNA strand exchange and ATPase function *in vitro*. Furthermore, it is shown that the inhibitory effect of PprA on DrRecA catalyzed DNA strand exchange was not due to sequestration of homologous dsDNA and was dependent on PprA oligomerization and DNA binding property. Together, results suggest that PprA is a new member of recombination mediator proteins (RMPs), and able to regulate the DrRecA function in γ-irradiated cells by protecting the *D. radiodurans* genome from hyper-recombination and associated negative effects.

## Introduction

An astounding gamma radiation resistance of *Deinococcus radiodurans* has been attributed to its efficient DNA double-strand break (DSB) repair supported by the Extended Synthesis Dependent Strand Annealing (ESDSA) mechanism and the ability to protect its biomolecules from oxidative damage (Zahradka et al., 2006; Slade et al., 2009; Misra et al., 2013). In bacteria, RecA plays an important role in homologous recombination (Bell and Kowalczykowski, 2016) and thus becomes integral to macromolecular events responsible for DNA strand break repair by homologous recombination and genome integrity (Heyer, 2015). RecA plays a deterministic role in both RecBC and RecF pathways of homologous recombination and several interacting proteins may involve in the regulation of RecA functions, viz. RecBC, RecF, RecO, RecR, DinI, RecX, RdgC, PsiB, and UvrD at multiple levels (Cox, 2007). In *Escherichia coli*, the expression of RecA is under the control of SOS regulon while the C-terminal region of RecA protein autoregulates its functions (Little and Mount, 1982). For bacterium *D. radiodurans*, DNA DSB repair and cell survival are heavily relying on RecA-mediated homologous (Daly et al., 1994; Daly and Minton, 1996; Zahradka et al., 2006; Slade et al., 2009). *D. radiodurans* lacks the LexA/RecA mediated canonical SOS regulation as DrRecA expression and/or activity is not under the control of either LexA proteins or its operon partners (CinA and LigT) (Narumi et al., 2001; Bonacossa de Almeida et al., 2002; Jolivet et al., 2006; Satoh et al., 2006). Nonetheless, two transcriptional regulators; IrrE and DdrO are shown to be a transcriptional regulator of *the recA* gene in the gamma-irradiated *D. radiodurans* cells (Earl et al., 2002), while DrRRA is shown to be a positive regulator during normal growth (Wang et al., 2008). Recently, phosphorylation mediated regulation of DrRecA function has been suggested, where it was shown that a radiation responsive RqkA kinase phosphorylates at Y77 and T318 amino acid and these sites phosphorylation has a substantial impact on nucleotide preference and DNA affinity of DrRecA (Rajpurohit et al., 2016) and the conformational stability, dynamics of DrRecA (Sharma et al., 2020). RecX of *D. radiodurans* is a negative regulator of *recA* expression as well could directly inhibit RecA activities like DNA strand exchange, ATPase activity, and LexA cleavage (Sheng et al., 2005). Interestingly, RecX does not seems to be a regulator of DrRecA under gamma radiation conditions as radiation resistance of *recX*^–^ mutant was similar to that of wild-type *D. radiodurans* (Sheng et al., 2005). *D. radiodurans* cells also lack many known RecA protein regulators (RecB, RecC, RecE, and RecT) as known for *E. coli* (Slade and Radman, 2011). Thus, it is likely that some new protein regulators or other novel mechanisms may regulate DrRecA activity in the gamma-irradiated cells.

PprA (Pleiotropic protein promoting DNA repair) is a unique DNA repair protein contributing to ionizing radiation and desiccation resistance as transcription of this gene induced multi-fold when *D. radiodurans* cells exposed to gamma radiation and by desiccation (Liu et al., 2003; Narumi et al., 2004). Biochemically, it stimulates ATP and NAD-dependent DNA ligases and protects DNA ends from exonucleolytic degradation (Narumi et al., 2004). It also binds with double-strand DNA ends to promote DNA looping (Adachi et al., 2014). The *pprA*^–^ mutant of *D. radiodurans* grows slowly and displays high sensitivity to UV-A radiation (Bauermeister et al., 2009), ionizing γ- radiation, and mitomycin C (Narumi et al., 2004). Interestingly, the γ-radiation sensitivity level of *the pprA*^–^ mutant is much lesser compare to the *recA*^–^ mutant. The γ-radiation survival of double mutant of *pprA* and *recA* genes (*pprA*^–^*recA*^–^) did not show additive sensitive phenotype (Tanaka et al., 2004), suggesting that both the proteins seem to contribute to radioresistance of *D. radiodurans* through common pathways and epistatic. However, the precise nature of *recA* and *pprA* interaction at a cellular and genetic level is not known and would be worth unveiling. Here, we report the physical and functional interaction of PprA with DrRecA and demonstrated that the role of PprA in the regulation of DrRecA biochemical properties and the recombination frequencies in the γ-irradiated *D. radiodurans*. The *pprA*^–^ mutant showed an increased recombination frequency in γ-treated cells and increased genomic instability in cells grown under normal conditions. Further, we showed that the inhibitory effect of PprA on DrRecA catalyzed DNA strand exchange activity was independent of PprA capability of sequestration of homologous DNA but dependent on PprA oligomerization and its DNA binding property. Together, results highlight the importance of PprA-DrRecA interaction in the regulation of DrRecA function under γ-irradiated conditions perhaps by protecting the genome from hyper-recombination and associated negative effect during post-irradiation recovery of *D. radiodurans.*

## Results

### PprA Protein Interacts With DrRecA

**FIGURE 1:**
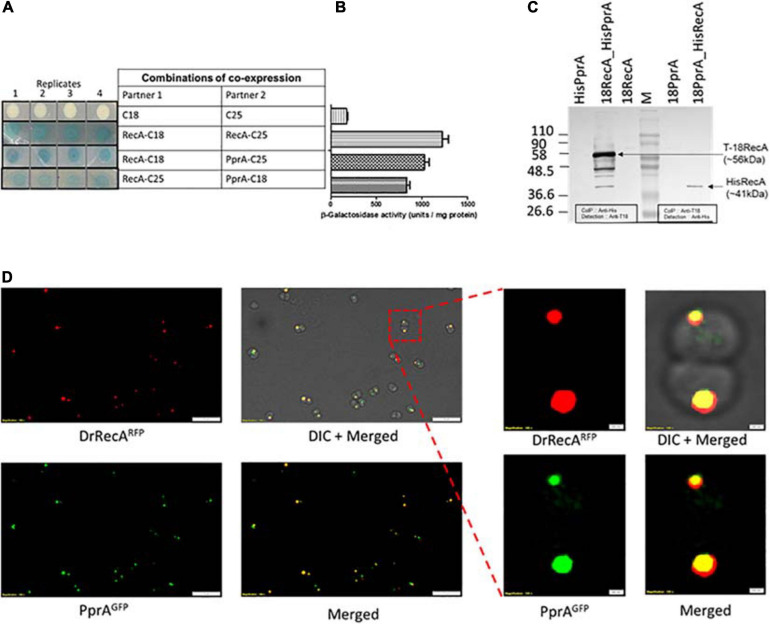
DrRecA and PprA interaction studies in surrogate *E. coli* and *D. radiodurans* cells. **(A,B)** T18 and T25 domains of adenylate cyclase were tagged with the PprA and RecA of *D. radiodurans* by cloning in BACTH plasmids. These plasmids were transformed into an *E. coli* BTH101 host. The interaction of proteins tagged with T18 and T25 were monitored as white-blue colonies **(A)** and β-galactosidase expression in liquid culture **(B)**. RecA-C18 and RecA-C25 were used as positive control while C18 and C25 tags expressing cells were used as a negative control. **(C)** Cell-free extracts of *D. radiodurans* cells co-expressing C18-RecA and His-PprA or C18-PprA and His-RecA from respective pVHSM and pRAD plasmid co-transformed in *D. radiodurans* cells, used for immunoprecipitation assay. An immunoprecipitation done using anti-His/anti-T18 antibody (Ab) and immunoprecipitates were separated on SDS-PAGE followed by immunoblot detection using antibodies against T18 domain of CyaA (anti-T18) or antibodies against histidine-tag (anti-His) antibody (Ab) as detailed in Materials and Methods. Data in panels **(A,C)** are representative of results from a reproducible independent experiment repeated three times, while data in panel **(B)** represent means ± SD (*n* = 9). **(D)** Fluorescence microscopy of *D. radiodurans* cells expressing DrRecA^RFP^ and PprA^GFP^ grown to logarithmic phase was carried out. The expression of DrRecA is visualized in the RFP channel (DrRecA^RFP^), GFP channel (PprA^GFP^), and under bright field (DIC). Merged images depict the colocalization of RFP and GFP fluorescence. Inset shows the single diplococci of *D. radiodurans* cells expressing DrRecA^RFP^ and PprA^GFP^ and their merged image along with DIC image. Scale and magnification are given in images.

PprA protein assists in DNA repair and cell survival of *D. radiodurans* recovering from ionizing radiation, and included in the DrRecA epistatic group (Narumi et al., 2004; Tanaka et al., 2004). The physical interaction of PprA and DrRecA protein was monitored using a bacterial two-hybrid system in surrogate *E. coli* BTH101, co-expressing T18 tagged PprA and T25 tagged DrRecA and also with tag swapped version of these proteins (Figure 1A). In the *E. coli* BTH101 cells, a functional gain of β-galactosidase activity due to interaction of tagged proteins (here DrRecA and PprA). The *in vivo* functional interaction of tagged proteins monitored as a function of β-galactosidase enzyme activity (Karimova et al., 2000). The nature and relative strength of the interaction between DrRecA-C18 and PprA-C25 was comparable to positive control where inter-subunit of RecA-RecA interaction was measured (Figures 1A,B). Similarly, in the tag swapped experiment, where RecA-C25 and PprA-C18 protein expressed in BTH cells, the interaction strength and β-galactosidase activity were comparable to DrRecA-C18 and PprA-C25 interaction results (Figures 1A,B). The β-galactosidase activity was minimal in the negative control, where T18 and T25 tags were expressed in BTH101 (Figures 1A,B).

The interaction of these proteins was also assayed in *D. radiodurans* by Co-immunoprecipitation (Co-IP) and by cellular co-localization. For Co-IP, DrRecA (poly His-tagged) and PprA (T18 tagged) were expressed either alone or together in *D. radiodurans* cells. Tag swap experiment was also done where DrRecA (T18 tagged) and PprA (poly His-tagged) together expressed in *D. radiodurans* cells (Supplementary Figure 1). Results showed that cells expressing alone DrRecA (T18 tagged) or PprA (poly His-tagged) did not produce a signal on blot when Co-IP was done with anti-His antibody (Ab) followed by detection by Anti-T18 Ab (Figure 1C). Similarly, in tag swapped experiment when PprA (T18 tagged) alone expressed and Co-IP was done with anti-T18 Ab followed by detection by Anti-His Ab (Figure 1C). The immunoprecipitate from cells expressing both proteins (T18 tagged DrRecA and poly His-tagged PprA) produce a band of molecular mass of ∼56 kDa (a theoretical size of T18 tagged DrRecA) Co-IP carried by anti-His antibody (Ab) and detection by Anti-T18 Ab (Figure 1C). The tag swapped experiment (poly His-tagged DrRecA and T18 tagged PprA) produces a band of molecular mass of ∼41 kDa (a theoretical size of poly His-DrRecA) (Figure 1C). Results of Co-IP data suggest that DrRecA and PprA interact with each other and could able to form a relatively stable complex which can be pulled down using Ab against one partner and the presence of an interacting partner in immunoprecipitant could be detected using Ab specific to another partner (Figure 1C). To further validate the interaction of DrRecA-PprA proteins *in solution*, the equimolar concentration of both proteins mixed and incubated for 10 min in the HEPES buffer followed by cross-linking of the interacting complex by glutaraldehyde and complex separated on SDS-PAGE. Results showed that both proteins form a stable complex and appeared a high molecular mass complex on SDS-PAGE gel (Supplementary Figure 2, PprA^wt^). Interestingly, though the presence of linear dsDNA induces the complex formation. However, later removal of DNA by DNAase treatment or using PprA mutant either lacking DNA binding (Supplementary Figure 2, PprA^R166A^) or defective in oligomerization (Supplementary Figure 2, PprA^R212A^) did not lose the ability of the physical interaction of proteins, together suggestive of physical interaction of DrRecA-PprA protein *in solution* which may further be supported by the presence of linear dsDNA (Supplementary Figure 2). Additionally, co-localization of DrRecA and PprA proteins was also ascertained by co-expressing of GFP-PprA and RFP-DrRecA fusion proteins in *D. radiodurans* cells and observation of fluorescence under a fluorescence microscope. The majority of cells expressing the GFP-PprA and RFP-DrRecA fusion proteins form definitive foci and a large number of foci showed colocalization in *D. radiodurans* cells (Figure 1D). Since both proteins were shown to be DNA repair proteins and showed cellular colocalization together support the observation of physical interaction of DrRecA and PprA protein in *D. radiodurans.*

### PprA Contributes to Recombination Frequency and Genetic Stability in *Deinococcus radiodurans*

Radiation resistance of *pprA*^–^ mutant and PprA overexpressing *D. radiodurans* cells were monitored (Supplementary Figure 3). As reported earlier, *pprA*^–^ mutant showed sensitivity to gamma radiation, but the overexpression of PprA in the wild type did not change its response to gamma radiation (Supplementary Figure 3). Although, DrRecA plays an important role in *D. radiodurans* resistance to genotoxic effects of radiation, desiccation, and oxidative stress (Jolivet et al., 2006; Schlesinger, 2007; Slade et al., 2009; Rajpurohit et al., 2016), its unregulated functions might result to hyper recombination leading to genomic instability in bacteria (Sander et al., 2003; Sheng et al., 2005).

**FIGURE 2:**
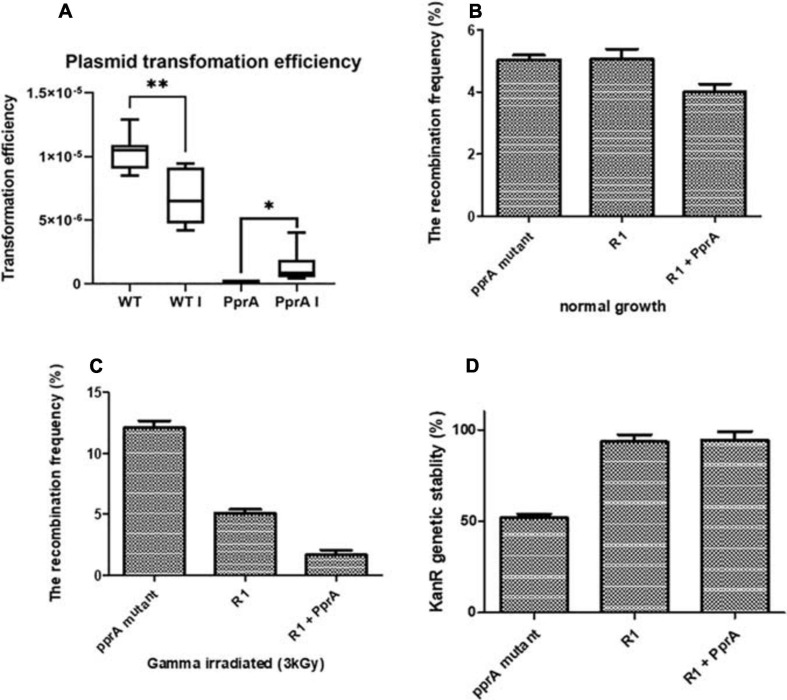
Transformation efficiency, recombination frequency, and genomic stability. **(A)** Plasmid pVHS559 was used to evaluate the transformation efficiency of γ-irradiated and unirradiated wild type and *pprA*^–^ mutant of *D. radiodurans* cells. Transformants were selected on an appropriate antibiotic. Transformation efficiently calculated by calculating CFU/μg plasmid DNA used. **(B,C)** Recombination frequency of *nptII* gene (confer kanamycin resistance) in **(B)** growing normally, and **(C)** γ-irradiated cells of wild type (R1), *pprA*^–^ mutant, and PprA over-expressed cells (R1 + PprA). **(D)** Genomic stability of nptII marker (confer kanamycin resistance) after 15th generations of wild type (R1), pprA- and PprA over-expressed cells (R1 + PprA). Data represented here is means ± SD (*n* = 9).

PprA protein could able to interact with DrRecA physically *in solution* and *in vivo* (Figure 1), therefore, we tested the role of PprA in the modulation of DrRecA functions in the normal and γ-stressed *D. radiodurans* cells. The effects of PprA on recombination and genomic stability were monitored by measuring the recombination frequency of the *nptII* gene (pNOKpqq plasmid confer Kan^R^) in either *pprA*^–^ mutant or PprA overexpressing *D. radiodurans* grew under normal and gamma stressed conditions (Figures 2B,C). Since the transformation efficiency of *pprA*^–^ mutant was ∼10-fold less than wild type *D. radiodurans* (Figure 2A), the recombination frequency normalized with transformation efficiency and normalized recombination frequency did not change significantly in both the *pprA*^–^ mutant or PprA overexpressing wild type cells grown under normal condition (Figure 2B). However, the irradiated (3kGy) *D. radiodurans* cells showed ∼10% normalized recombination frequency in the absence of *the pprA* gene while this frequency is ∼5% in the wild type and ∼1% in the cells overexpressing PprA (Figure 2C). These results suggested that the presence of PprA protein in cells could negatively regulate the recombination in gamma-irradiated cells *in vivo* and cells lacking *the pprA* gene showed relatively higher recombination events compare to wild-type cells.

Genomic instability was also tested by scoring the kanamycin resistance of cells of different genetic backgrounds from the above experiment. A significant loss of kanamycin resistance gene (*nptII*) (∼50%) was observed in the *pprA*^–^ mutant after the 15th generation of growth, while it did not change in the wild-type cells overexpressing PprA and was similar to wild type control (Figure 2D). This result emphasizes that the presence of PprA protein helps in maintaining the genomic stability in *D. radiodurans* cells while in *pprA*^–^ mutant, the kanamycin resistance has lost progressively over the generation possibly due to genomic instability. Collectively, results about recombination and genomic stability suggested that the absence of the *pprA* gene alleviates the recombination frequency in the gamma-irradiated *D. radiodurans* cells and also impacts the genomic stability of the marker gene (*nptII*) in the cells growing normally.

### PprA Inhibit Strand Exchange Promoted by DrRecA

**FIGURE 3:**
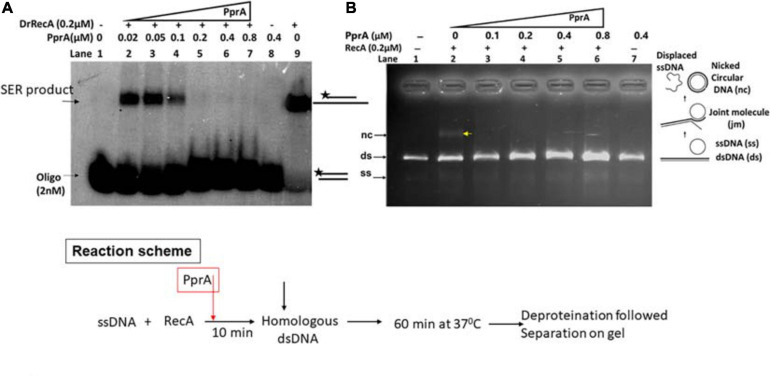
Inhibitory effect of PprA protein on DrRecA catalyzed DNA strand exchange. **(A)** Oligo-based DNA strand exchange catalyzed by DrRecA and **(B)** DrRecA catalyzed DNA strand exchange between M13mp18 ssDNA and linear dsDNA. In both the experiments, an increasing concentration of PprA protein was added in the reaction to see the inhibitory effect of PprA protein on DrRecA function as detailed in methods.

The recombination events in gamma-irradiated *D. radiodurans* cells had increased significantly in *pprA*^–^ genetic background (Figure 2C) suggestive of the possible inhibitory effects of PprA interaction on DrRecA functions *in vivo*. Therefore, the effect of PprA on recombination activity of DrRecA was examined in an assay using short (40 bp) and long homology (M13 DNA) at varying concentration of PprA (0.02–0.8 μM) and a fixed concentration of DrRecA (0.2 μM) as reaction scheme given in Figure 3. Results showed significant inhibition of heteroduplex formation with increasing concentration of PprA protein (Figure 3A, lane 2–7 and Figure 3B, lane 3–6). It may be noted that equimolar concentration of PprA (0.2 μM) could exert the maximum inhibitory effect (Figure 3A, lane 5 and Figure 3B, lane 4). PprA alone did not catalyze the heteroduplex formation (Figure 3A, lane 8 and Figure 3B, lane 7), while DrRecA without PprA showed an efficient DNA strand exchange activity (Figure 3A, lane 9 and Figure 3B, lane 2). These results together prove the inhibitory effect of PprA protein on DrRecA promoted DNA strand exchange reaction.

### The Inhibitory Effect of PprA Protein Is Not Due to Sequestration of Homologous dsDNA During DNA Strand Exchange

**FIGURE 4:**
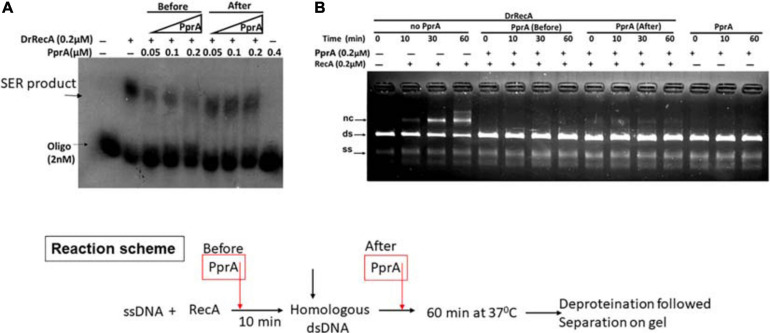
Inhibitory effect of PprA protein when added before and after addition of dsDNA. An oligo and long homology M13-based DNA strand exchange assay was employed to see the inhibitory effect of PprA protein. **(A)** In oligo-based strand exchange increasing concentration of PprA protein (0.05–0.2 μM) added to the reaction mixture as mentioned in methods before and after the addition of dsDNA 40-mer and product formation visualized by autoradiogram. **(B)** M13mp18-based strand exchange reaction (SER) where reaction carried out as stated in methods without adding PprA (no PprA), the addition of PprA before M13 linear dsDNA (PprA before) or 0.2 μM PprA addition after M13 linear dsDNA (PprA after). As a control reaction 0.2 μM PprA protein was added without the addition of DrRecA protein. all reactions were incubated till 60 min and samples were drawn at the indicated time and separated on 0.8% agarose gel after deproteinization.

**FIGURE 5:**
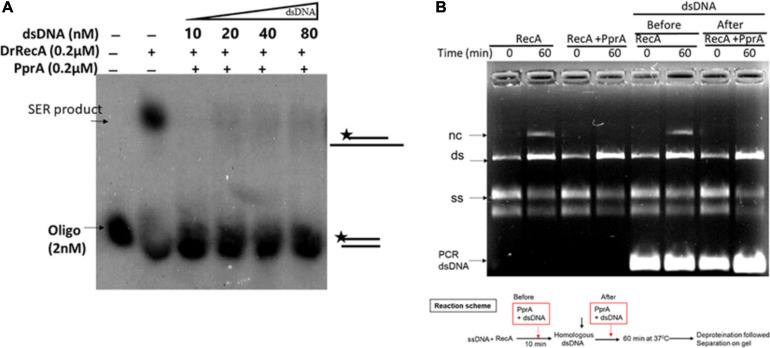
PprA protein led inhibition of DrRecA DNA strand exchange is not due to sequestration of homologous DNA. **(A)** PprA protein was added to the reaction along with molar excess of dsDNA (5–40-fold). **(B)** SER reaction performed using M13 substrates, and addition of five molar excess of 1 kb non-specific dsDNA in the reaction after addition of specific dsDNA did not rescue the SER product formation, when PprA protein present. The products were analysed and visualized on gel and autoradiogram.

To understand a mechanistic aspect of the PprA inhibition of DrRecA catalyzed DNA strand exchange reaction (SER), the SER assay was performed as discussed above and the PprA protein was added before and after the addition of homologous dsDNA (Figure 4). The pre-incubation of PprA with DrRecA led to complete inhibition of recombination reaction (Figures 4A,B). However, the inhibition was significantly less when PprA was added after the addition of dsDNA (Figure 4). A similar effect was observed in the strand exchange assay with a long homology substrate (Figure 4B). In the absence of PprA, DrRecA could efficiently catalyzed the reaction and recombinant product formation (Figures 4A,B). However, PprA addition after dsDNA addition in the reaction showed ∼70% substrate conversation to product compare to reaction lane where PprA did not add (Figure 4). The observation of the complete inhibition of recombinant product, when PprA protein added before dsDNA addition allows us to speculate that PprA physical interaction with DrRecA may affect DrRecA ability to either interact ssDNA or RecA polymerization during the formation of presynaptic filament. PprA is a non-specific dsDNA binding protein and has nearly negligible affinity for ssDNA (Narumi et al., 2004; Rajpurohit and Misra, 2013; Adachi et al., 2014). Thus, the possibility of homologous dsDNA sequestration by PprA might affect the strand exchange activity of DrRecA was examined in the presence of 5–40-fold molar excess of non-specific dsDNA (Figure 5). The addition of an increasing concentration of non-specific dsDNA did not rescue DrRecA strand exchange activity from PprA led inhibition (Figure 5A). A similar observation was also confirmed using M13 substrates, where strand exchange reaction was performed in the presence of five molar excess of 1 kb non-specific dsDNA (Figure 5B). These findings together highlight the direct inhibitory effect of PprA on DrRecA catalyzed SER, however, this effect is not due to limiting the availability of homologs DNA by PprA during DNA strand exchange reaction.

### PprA Protein Hamper the ssDNA Stimulated ATPase of DrRecA

**FIGURE 6:**
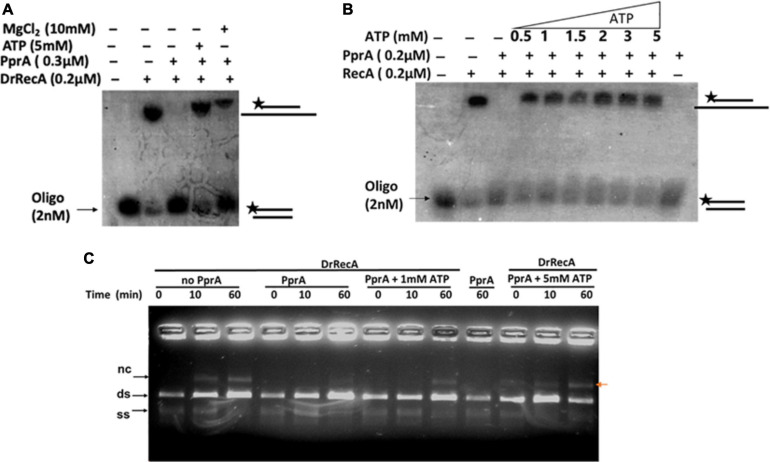
Rescue effect of ATP and Mg^2+^ ion on PprA protein led inhibition of DrRecA DNA strand exchange. **(A)** 5 mM ATP and 10 mM MgCl_2_ were added to overcome the inhibitory effect of PprA in oligo-based DNA strand exchange reaction. **(B)** Increasing concentration of ATP (0.5–5 mM) is used to counter the inhibitory effect of PprA in reaction. **(C)** 1 and 5 mM ATP addition in M13mp18-based DNA strand exchange reaction proves to be effective to counter PprA inhibitory effect. Whereas, the addition of PprA without the addition of additional ATP inhibits DrRecA function in DNA strand exchange.

**FIGURE 7:**
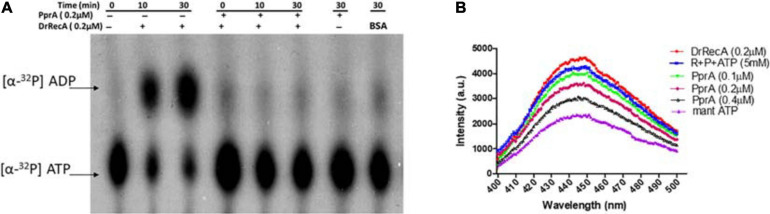
Inhibitory effect of PprA protein on DrRecA ATPase and ATP binding. **(A)** The inhibitory effect of PprA protein on DrRecA ATPase was assayed by thin-layer chromatography (TLC). The hydrolysis of [α^−32^P] ATP to [α^−32^P] ADP monitored on a TLC plate followed by an autoradiogram. **(B)** ATP binding assay of DrRecA (0.2μM) was monitored with fluorescent ATP (Mant ATP) in the absence (DrRecA) and presence of PprA protein (0.1 to 0.4μM) separately and together with DrRecA + PprA + ATP (R + P + ATP). Data was recorded on Spectro-fluorimeter in the range of 400 to 500nm wavelength.

How PprA inhibits the DrRecA catalyzed SER is not clear and hypothesized that PprA interaction with DrRecA may hamper either DrRecA functional biochemical activities such as access to DNA substrates, ATPase activity, or PprA may limit metal ion availability to DrRecA. PprA protein did not show binding with ssDNA, however able to bind with dsDNA and form a distinct DNA-PprA nucleoprotein complex than the DNA-DrRecA nucleoprotein complex (Supplementary Figure 4). Interestingly, PprA did not limit the access of DrRecA to DNA substrates as the binding of DrRecA to ssDNA and dsDNA with and without PprA protein was found to be similar (Supplementary Figure 4). This observation was further supported by data presented in Figure 5, where the addition of molar excess non-specific dsDNA did not rescue the inhibitory effect of PprA (Figure 5). Next, we checked the possibilities of limiting the availability of ATP or metal ion by PprA when added in DrRecA catalyzed SER. For that, an assay was performed where PprA protein-mediated inhibition of DrRecA catalyzed DNA strand exchange (short homology oligo-based) was rescued by adding molar excess of ATP and metal ion (MgCl_2_) (Figure 6A). Data showed in Figure 6A suggested that the addition of 5 mM ATP could able to restore the strand exchange product formation in the presence of PprA while excess metal ion (10 mM) did not restore the reaction (Figure 6A). A similar observation was also apparent in the M13 based SER (Figure 6B). No adverse effect of excess ATP (5 mM) was observed in DrRecA catalyzed strand exchange (Supplementary Figure 5). Collectively, these data conclude that PprA did not limit the binding of DrRecA to DNA as well as the availability of metal ions rather PprA may have an effect on either limiting the ATP hydrolysis or availability to DrRecA during strand exchange reaction as the addition of ATP (1–5 mM) could effectively reverse the inhibitory effect of PprA (Figures 6B,C). To address these possibilities, ssDNA-dependent ATPase activity of DrRecA was checked in the presence and absence of equimolar concentration of PprA protein. Results showed that DrRecA display strong ssDNA-dependent ATPase activity (Figure 7A). The addition of equimolar concentration of PprA protein resulted in strong inhibition of DrRecA ATPase and could not be restored till 30 min of reaction time (Figure 7A). PprA alone or BSA (negative control) did not hydrolyze the ATP (Figure 7A). The experiment results reveal the mechanistic proof of PprA protein led inhibition of DrRecA by limiting the DrRecA ATPase function. This observation was further corroborated by a fluorescent ATP (mant-ATP, sigma) binding assay. In this assay, binding of fluorescent ATP by DrRecA was found to be inhibited by increasing the concentration of PprA protein (0.1–0.4 μM) and this inhibition could be reversed by the addition of ATP (5 mM) (Figure 7B). Together, presented data suggested that PprA could impede the DrRecA by either sequestering the ATP in solution or by limiting ATP access to the nucleotide-binding pocket of DrRecA and consequently interfering with the ATP hydrolysis. Since PprA was not able to bind and hydrolyze ATP (Figure 7A), the possibilities of DrRecA’s ATPase inhibition by PprA protein would possibly due to the inability of DrRecA filament to either bind or hydrolyze the ATP when it forms physical interaction with PprA.

### PprA Mutants Lacking Oligomerization and DNA Binding Could Not Inhibit the DrRecA

**FIGURE 8:**
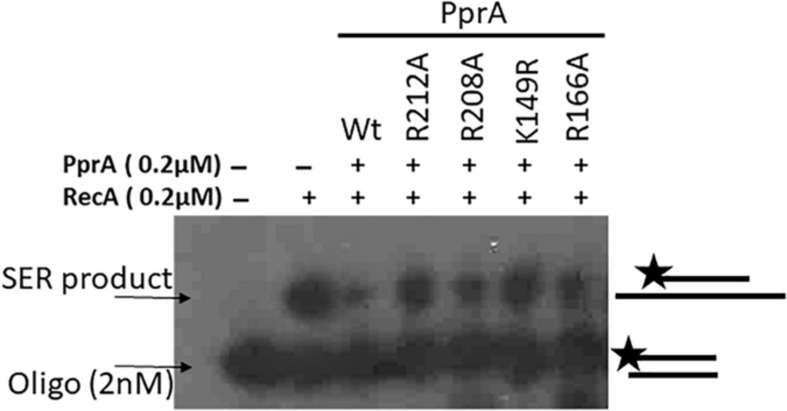
An inhibitory effect of PprA and its mutants on SER of DrRecA. The inhibitory effect of PprA and its mutants on SER of DrRecA was evaluated using an oligo-based DNA in the presence of PprA and its DNA binding (K149A, and R166A) and oligomerization (R208A, and R212A) mutants. Products were analyzed on gel and visualized on autoradiogram.

PprA protein has both dsDNA binding and oligomerization properties (Narumi et al., 2004; Rajpurohit and Misra, 2013; Adachi et al., 2014). Our interest was to find out how do these properties contribute to PprA inhibition of DrRecA function. Previously, R208A and R212A mutants of PprA lacking both oligomerization and DNA binding properties, while K149A and R166A mutants lacking DNA binding activity but proficient in oligomerization were reported (Adachi et al., 2014). We have generated these mutants and their properties were verified and found to similar to as reported earlier (data not shown). The strand exchange reaction was monitored in the presence of PprA and its R208A, R212A, K149A, and R166A mutants Interestingly, all four mutants either defective in DNA binding activity (R208A, R212A, K149A, and R166A) or oligomerization (R208A and R212A) showed marginal inhibition on DrRecA catalyzed DNA strand exchange reaction (Figure 8), while wild type PprA having intact DNA binding and oligomerization properties was being able to efficiently inhibit DrRecA functions (Figure 8). Interestingly, DNA binding mutant (R166A) and oligomer mutant (R212A) of PprA retained their ability to interact with DrRecA similar to wild-type PprA (Supplementary Figure 2). However, these mutations of PprA protein (R166A and R212A) hamper their ability to interfere with DrRecA catalyzed SER raised the possibility of a more dynamic nature of the interaction of PprA interaction with DrRecA. Nonetheless, these results highlighted the crucial role of PprA DNA binding and oligomerization ability in the inhibition of DrRecA function *in vitro*.

## Discussion

*Deinococcus radiodurans* cells have an extraordinary DNA repair capability and can endure a high level of genetic perturbation caused by ionizing radiation, desiccation, and stress-induced by cold conditions (Cox and Battista, 2005; Slade et al., 2009; Misra et al., 2013). DrRecA mediated recombination repair required for radiation-resistant phenotype and *recA* mutant of *D. radiodurans* highly sensitive to gamma radiation, UV radiation, and MMC (mitomycin C) treatment (Moseley and Copland, 1975; Gutman et al., 1994; Rajpurohit et al., 2016). Moreover, radiation sensitivity directly correlated with reduced recombination frequency in *recA* mutant of *D. radiodurans* (Moseley et al., 1972; Daly et al., 1994; Daly and Minton, 1996). RecA and its homolog catalyzes homologous recombination repair (HRR) of the collapsed replication fork, DNA DSBs, and involve in the maintenance of genomic integrity (Li and Heyer, 2008). The regulation of bacterial RecA function is highly diverse and is regulated by many proteins. The regulatory proteins catalog affecting the function of bacterial RecA is increasing and many new candidates have been added to this list in the recent past (Cox, 2007). To add a new candidate to this list, the present study has brought forth a PprA protein as a negative regulator of DrRecA. In bacteria, canonical mechanisms of RecA regulation; is SOS regulatory mechanism and the same was found to be redundant in the case of regulation DrRecA (Narumi et al., 2001; Slade et al., 2009). Therefore, some new mechanisms that could regulate DrRecA expression have been suggested (Earl et al., 2002; Wang et al., 2008; Devigne et al., 2015; Blanchard et al., 2017). Phosphorylation-mediated regulation of DrRecA activity and structure dynamics has been recently shown (Rajpurohit et al., 2016; Sharma et al., 2020). *D. radiodurans*, RecFOR proteins that help the loading of RecA on DNA substrate were shown to be crucial for DrRecA function (Slade et al., 2009; Bentchikou et al., 2010), while RecX is shown to be a negative regulator of DrRecA and causes net disassembly of RecA nucleoprotein filament through physical interaction and mitigating the possibilities of hyper recombination that would be deleterious for the genome integrity under normal growth of this bacterium (Sheng et al., 2005). The present study has provided evidence to suggest the regulatory role of PprA protein in the regulation of DrRecA functions and possible underlying mechanisms to explain the epistatic natures of *pprA* and *recA* genes in *D. radiodurans*, particularly in γ-irradiated cells. The data presented in this study supported the following conclusions, (1) DrRecA and PprA protein interact physically, (2) PprA role is crucial in minimizing the deleterious effect of DrRecA due to possible hyper recombination activity in the cells recovering from gamma irradiation and for the genomic stability of the cells growing normally, (3) PprA protein could interfere DrRecA catalyzed strand exchange reaction is due to impediment of the ATPase function of DrRecA, but not due to sequestration of homologous dsDNA, and (4) PprA The oligomerization and DNA binding properties crucial for PprA led inhibition of DrRecA function.

PprA and DrRecA could physically interact *in vitro* and *in vivo* (Figure 1). This observation is not surprising as the epistatic nature of these protein and the ability of PprA protein to interact with other DNA metabolic protein (DNA ligase, DNA gyrase, and topoisomerase IB) and DNA replication related proteins (DnaA and DnaB) due to its pleiotropic functions (Kota and Misra, 2008; Devigne et al., 2015; Maurya and Misra, 2020). The presence of dsDNA further augments the interaction of both proteins (Supplementary Figure 2) and was further supported by the inability of PprA mutants (lacking DNA binding and oligomerization properties) to exert an inhibitory effect on DrRecA catalyzed SER (Figure 8). The requirement of intact DNA binding and oligomerization properties of PprA protein for the maximum inhibitory effect on DrRecA catalyzed DNA strand exchange (Figure 8) is intriguing and raises the possibility that DNA might function as a mediator for this interaction. PprA protein has dsDNA binding properties but lacking ssDNA binding (Adachi et al., 2014), while DrRecA has both ssDNA and dsDNA binding properties with more affinity toward dsDNA in the absence of nucleotide cofactor (Warfel and LiCata, 2015; Rajpurohit et al., 2016; Sharma et al., 2020). The binding of DrRecA to ssDNA and dsDNA was least affected by the presence of an equimolar concentration of PprA (Supplementary Figure 4). Thus, it is likely that PprA may suppress the DrRecA activity by possible interdependent mechanisms; where direct binding of PprA to DrRecA may have further augmented by DNA binding ability of PprA protein to make a stable complex with DrRecA (Figure 8 and Supplementary Figure 4). RecA promoted DNA strand exchange reaction begins with the loading of RecA on the single-stranded DNA (ssDNA) to form nucleoprotein filament which searches for homologs double-strand DNA (dsDNA) and facilitate the strand exchange (Shan and Cox, 1997; Yu et al., 2001). Interestingly, ATP hydrolysis is not required for the formation of heteroduplex complex during DNA strand exchange reaction as RecA may able to perform a search for homologs DNA even in the presence of a non-hydrolyzable ATP analog; ATPγS or in the presence of ADP-AlF4 analog (Menetski et al., 1990). Therefore, it was proposed that RecA unsaturated nucleoprotein filament propel the DNA exchange until the newly formed heteroduplex molecule keeps releasing from triple-helix nucleoprotein complex during SER (strand exchange reaction) and this function is being facilitated by ATP hydrolysis. Therefore, ATP hydrolysis by nucleoprotein filament is crucial for propelling the strand exchange reaction in the forward direction (Kowalczykowski et al., 1987). The established hypothesis about RecA mediated DNA strand exchange suggest that nucleoprotein filaments adopt a stretched, rigid, under-wound B-DNA-like conformation (Chen et al., 2008), and the discontinuities in RecA nucleoprotein filaments would terminate the strand exchange and start homology search (Shan and Cox, 1997). Thus, ATP hydrolysis by RecA nucleoprotein filament offers dynamics to the RecA nucleoprotein filaments (van Loenhout et al., 2009). Data from the present study suggest that PprA protein interaction with DrRecA causes severe inhibition of ATPase function of DrRecA nucleoprotein filament (Figure 7) and inhibition of DNA strand exchange (Figure 3). The PprA led inhibition of DrRecA strand exchange was could not be rescued by adding molar excess of dsDNA or metal ion suggest that the inhibitory effect of PprA is not indirect rather through direct interaction with DrRecA filament and inhibition of ATPase function. The exact mechanism and the nature of this interaction are not clear but PprA interaction with DrRecA nucleoprotein filament interaction may likely either freeze the domain motion of nucleoprotein filament of DrRecA assisted by its ATPase activity or may limit the access the ATP to DrRecA nucleoprotein. Since PprA alone did not have ATPase and neither it can bind with ATP, thus former possibility is more likely. Recently, the functional implications of RecA unsaturated and saturated nucleoprotein filaments formation and the role of RecA ATPase function to regulate the dynamic equilibrium was probed by Zhao et al. (2017) by capillary electrophoresis-laser-induced fluorescence polarization assay (CE-LIFP) and suggest that RecA unsaturated nucleoprotein filaments predominate under physiologically relevant conditions over long saturated RecA nucleoprotein filaments and these unsaturated nucleoprotein filaments are key driver scaffolds for the DNA strand exchange and homologous recombination (Zhao et al., 2017). Therefore, ATPase function is not only required for the removal of RecA from heteroduplex complex but also facilitates the formation of unsaturated nucleoprotein filament continuously to propel the DNA strand exchange reaction in a forward direction (Kowalczykowski et al., 1987; Zhao et al., 2017). The ATPase function of DrRecA nucleoprotein filaments may exist in an inactive default state under the condition when protein is bound to dsDNA. However, the inactive state changes to an active ATPase state when ssDNA is added to the reaction or in the presence of lower pH or by volume exclusion agents (Ngo et al., 2013). In general, the ATPase function of RecA gives mechanical power for nucleoprotein filament remodeling and dysfunctional ATPase would hamper the remodeling capacity of RecA filament and resultant no strand exchange product will be formed. Thus, data presented in the present study suggested that inhibition of ATPase of DrRecA by PprA may directly lead to an imbalance in DrRecA saturated and unsaturated nucleoprotein filament and resultant inhibition of DNA strand exchange.

**FIGURE 9:**
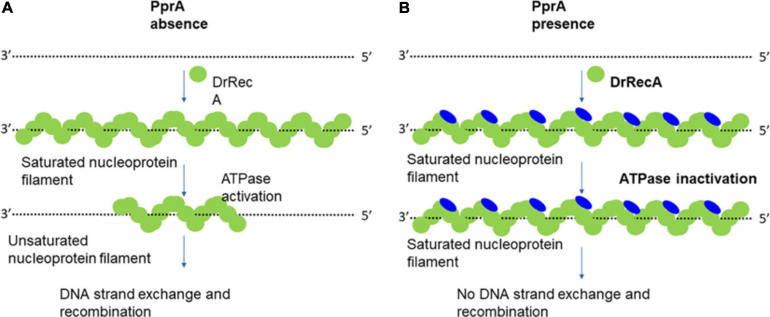
Model explains the working hypothesis of PprA protein-mediated inhibitory effect on DrRecA functions. **(A)** In the absence of PprA protein, DrRecA protein forms nucleoprotein filament on ssDNA. This RecA saturated nucleoprotein filament (inactive filament) converted to unsaturated nucleoprotein filament (active filament) by ATPase function of DrRecA. This dynamics facilitates the homology search by DrRecA for a successful SER. **(B)** PprA interaction with DrRecA inhibits its ATPase function of DrRecA nucleoprotein filament by either interfering with ATP binding that affects the dynamics of active and inactive filament nucleoprotein filament interchange and impairment of SER activity of DrRecA.

The activity of RecA is supposed to be highly regulated because unregulated RecA function may lead to hyper-recombination situations and could be deleterious for cell survival. Therefore, numerous protein regulator (RecBCD, RecFOR, SSB, LexA, UmuD, DinI, PsiB, RdgC, and RecX proteins) known to regulate bacterial RecA activity (Lusetti et al., 2004, 2006; Drees et al., 2006; Spies and Kowalczykowski, 2006; Cox, 2007). Here our data suggest that the PprA protein of *D. radiodurans* is a new regulator of RecA function, especially in the irradiated cells. Earlier it was shown that DrRecA could catalyzes the DNA strand exchange through unique inverse strand exchange and able to complement the RecA functions in *E. coli.* However, *E. coli* RecA could only complement partially the DrRecA functions suggesting that RecA regulatory network operates in *D. radiodurans* are different from *E. coli* (Narumi et al., 1999). Interestingly, the existence of PprA protein was reported only in the *Deinococcaceae* family, and no homolog was reported outside this family (Narumi et al., 2004), suggesting that RecA activity regulation by PprA protein may be limited to the *Deinococcaceae* family. However, it would be interesting to see the inhibitory effect of PprA on RecA from other bacteria like *E. coli*. The ectopic expression *pprA* gene in *E. coli* induces the catalase function and oxidative stress resistance but its interaction with *E. coli* RecA not studied (Kota and Misra, 2006). How does PprA precisely contributes to the regulation of recombination repair and DrRecA function in *D. radiodurans* needs further careful and thorough study. Nonetheless, the present study hitherto brought forth interesting observations about the negative regulation of DrRecA activities and recombination by PprA. The recombination frequency increases in *pprA*^–^ mutant cells after irradiation but, little change in recombination compare to wild-type cells in PprA overexpressing cells after irradiation could be due to very high overexpression *pprA* gene itself in wild type cells after irradiation (Figure 2). On the mechanistic front, we propose that DrRecA activity inhibition by PprA by impeding the ATPase function of DrRecA and altered nucleoprotein filament function which effectively diminishes the homology search and DNA strand exchange function of DrRecA (Figure 9). Zahradka et al. (2006) showed that DNA repair in *D. radiodurans* follows biphasic repair kinetics following exposure to extreme radiation, in phase I, massive DNA synthesis followed by assembly of DNA fragments occurs, which is dependent on DNA polymerase I activity and termed extended synthesis-dependent strand annealing (ESDSA) repair (Zahradka et al., 2006). Though the DrRecA level increased in the ESDSA phase, its function was primarily required in the later stage of repair where DrRecA mediated homologous recombination using substrate from ESDSA repair to produces full-length chromosomes (Liu et al., 2003; Slade and Radman, 2011). We believe that the implications of our finding of DrRecA and PprA interaction may help in allowing ESDSA repair by minimizing the DrRecA induced recombination events during ESDSA repair after acute doses of γ-radiation (Zahradka et al., 2006; Slade et al., 2009). Together, based on data presented here allow us to speculate that even though PprA work as an inhibitor of DrRecA by impeding its ATPase function but this inhibitory effect of PprA protein may well help *D. radiodurans* cells to efficiently repair shattered genome with the highest precision and thus help in maintaining genomic integrity.

## Materials and Methods

### Bacterial Strains, Growth Medium, and Plasmids

Wild type bacterium *D. radiodurans* R1 used from lab stock (ATCC 13939). *pprA*^–^ mutant was a generous gift from I. Narumi, Japan (Narumi et al., 2004). Wild type *D. radiodurans* and its mutant were gro*wn* in TGY medium (1% Bacto tryptone, 0.1% glucose, 0.5% yeast extract) with appropriate antibiotic as described earlier (Rajpurohit and Misra, 2010). For the cloning and maintenance of plasmids; *E. coli* Novablue strain was used while *E. coli* BTH101 (lacking cyaA, referred here as BTH101) was used for the coexpression of cloned proteins on BACTH plasmids for *in vivo* protein-protein interaction studies and grown at 30°C (Maurya et al., 2018). pUT18, pKNT25, and pET28a (+) plasmids and their derivatives were maintained in *E. coli* cells (Nova blue) in the presence of the required antibiotics. Shuttle vector for *E. coli* and *D. radiodurans* pVHS559 and their derivatives were maintained in the presence of spectinomycin *D. radiodurans* (70 μg/ml) and *E. coli* (150 μg/ml) (Maurya et al., 2018). Standard molecular biology techniques were used as described (Green and Sambrook, 2012). Antibodies against the T18 (SC-13582) and T25 (SC-33620) domains of CyaA of Bordetella pertussis were procured commercially (Santa Cruz Biotechnology, Inc.), an antibody (Ab) against Anti-His purchased from New England Biolabs (United States). Molecular biology grade chemicals, enzymes, and other salts used in this study were procured from different manufactures like Sigma Chemicals Company, United States; Roche Biochemicals, Mannheim, Germany; New England Biolabs (United States); and Merck India Pvt. Ltd., India. Radiolabeled nucleotides were obtained from the Board of Radiation and Isotope Technology (BRIT), Department of Atomic Energy, India. All the bacterial strains and plasmids used in this study are listed in Supplementary Table 1.

### Construction of Recombinant Plasmids and Protein Purification

List of plasmids and primers used in this study given in Supplementary Table 1. The transnational fusion of DrRecA and PprA protein with T18 tag and T25 tag obtained by cloning of coding sequence of *recA* and *pprA* gene in pUT18 and pKNT25 plasmids at the restriction sites indicated in Supplementary Table 1. Obtained plasmids for *recA* gene (pUTDr*recA* and pKNDr*recA*) and *pprA* gene (pUT*pprA* and pKNDr*pprA*) were transformed to *E. coli* BTH101. Coding sequences of polyhistidine-tagged DrRecA and PprA were PCR amplified using pETHisFw and pETHisRw primers from their respective pET28a (+) clones as a template (Kota and Misra, 2006; Rajpurohit et al., 2016), and were sub-cloned in shuttle plasmid pRADgro at *Apa*I and *Xba*I sites, yielding pRadHisrecA and pRadHispprA respectively. Similarly, the T18-tagged *recA* gene was PCR amplified using primers (BTH*recA*-F and BTH*recA*-R) and T18-tagged *pprA* genes using primers (BTH*pprA*-F and BTH*pprA*-R) and cloned in pVHS559 shuttle vector at *Nde*I-*Xho*I sites for coimmunoprecipitation studies in *D. radiodurans* (Supplementary Table 1). Expression of all fusion proteins was confirmed by Western blotting using antibodies against the T18 domain of C18-tag and polyhistidine-tag (Supplementary Figure 2). pVHpprA^GFP^ expressing PprA-GFP fusion protein constructed earlier and used here (Kota et al., 2014). For the construction of the DrRecA-RFP expression plasmid, the coding sequence of DrRecA was cloned at pDSred plasmid (Clontech) at *Kpn*I and *Bam*HI sites, yield pDSrecA^RFP^. The rfp–recA region was PCR amplified from pDSrecA and cloned at *Apa*I and *Eco*RV sites in pRAD plasmid and pRADrecA^RFP^ plasmid was obtained. Both pRADrecA^RFP^ and pVHpprA^GFP^ plasmids were transformed into *D. radiodurans* transformants were screened on TYG agar plates supplemented with spectinomycin (75 μg/ml) and chloramphenicol (7 μg/ml). Recombinant GFP-PprA was expressed by inducing the culture with 10 mM IPTG in the case of *D. radiodurans* while RFP-DrRecA expresses constitutively.

Recombinant plasmids pET*recA* and pET*pprA* used in this study were constructed earlier and described previously (Rajpurohit and Misra, 2013; Rajpurohit et al., 2016). Recombinant DrRecA and PprA were expressed in *E. coli* BL21(DE3) pLysS. Both proteins were purified as described previously (Rajpurohit and Misra, 2013; Rajpurohit et al., 2016). In brief, *E. coli* BL21(DE3) pLysS expressing recombinant proteins were harvested after 3 h post-induction by IPTG. The cell pellet was suspended in buffer A (20 mM Tris–HCl; pH 7.6, 150 mM NaCl) containing 10 mM imidazole, 0.5 mg/ml lysozyme, 1 mM phenylmethylsulfonyl fluoride (PMSF), 0.2% Triton X-100, and 10% glycerol and incubated at 37°C for 30 min. A protease inhibitor cocktail was added to the reaction mixture, and the cells were sonicated for 10 min using 5-s pulses with intermittent cooling for 10 s at 35% amplitude. The cell lysate was centrifuged at 12,000 rpm for 30 min at 4°C. The cell extract was loaded onto a NiCl_2_ charged-fast-flow-chelating Sepharose column (GE Healthcare) equilibrated with buffer A (20 mM Tris–HCl; pH 7.6, 300 mM NaCl, 10% glycerol). The column was washed with 20 column volumes of buffer A containing 20 mM imidazole until proteins stopped coming from the column. Recombinant proteins were eluted with buffer A containing 250 mM imidazole. Fractions were analyzed by SDS-PAGE, and those containing nearly pure proteins were pooled and their his-tag removed by incubating proteins with Factor-Xa (NEB). Untagged protein comes out in flow-through when loaded on Ni-NTA agarose column following the protocols described by the manufacturer (Qiagen, Inc.). Unbound proteins were further purified on Q-sepharose, Heparin, and Superdex-200 column. Proteins fractions free from detectable nuclease contamination and has more than 95% purity, were polled and precipitated by ammonium sulfate precipitation followed by dialysis in buffer B; 10 mM Tris–HCl (pH7.6), 50 mM KCl, 50% glycerol, and 1 mM PMSF and stored at −20°C Proteins.

### Protein-Protein Interaction Studies, Western Blotting, and Coimmunoprecipitation

A bacterial two-hybrid system (BACTH) is employed to ascertain the *in vivo* protein-protein interaction in *E. coli* as detailed elsewhere (Battesti and Bouveret, 2012; Siddiqui et al., 2017). BTH101 *E. coli* cells were transformed with different plasmids expressing target proteins with T18 tags or T25 tags at the C-terminus of target proteins, respectively. Empty vectors in BTH101 cells used as controls. The cells in quadruplet spotted on LB agar plates containing 5-bromo-4-chloro-3-indolyl-β-D-galactopyranoside (X-Gal) (40 μg/ml), IPTG (0.5 mM), and antibiotics as required. After overnight incubation of plates at 30°C, the appearance of white-blue colored colonies was recorded. In parallel, an aliquot of the same culture was grown overnight with 0.5 mM IPTG and appropriate antibiotics, and β-galactosidase activity was measured from liquid cultures as described earlier (Maurya et al., 2018). In brief, diluted culture (1:4) into LB medium with OD_600_ normalized. Cultures (100 μl) were mixed with 1 μl Z-buffer (60 mM Na_2_HPO_4_, 40 mM NaH_2_PO_4_, 10 mM KCl, 10 mM MgSO_4_, 50 mM β-mercaptoethanol, pH 7.0) followed by the addition of, 0.01% SDS and 20 μl chloroform to permeabilize the cells, and cell debris was removed. Enzyme activity was measured in triplicate with 50 μl of supernatant using 0.4% *O*-nitrophenyl-β-D-galactopyranoside (ONPG) as a substrate. The β-galactosidase activity was calculated in Miller units as described previously (Battesti and Bouveret, 2012). The interaction of DrRecA and PprA proteins *in solution* was assayed by a glutaraldehyde-assisted cross-linking experiment. In brief, both proteins (approx. 5 μg each) were mixed in HEPES buffer (pH 7.6) and allowed to interact for 10 min followed by addition of 0.5% glutaraldehyde added and reaction incubated for another 10 min reaction terminated by adding 2X SDS dye and samples analyzed on SDS-PAGE. 1 kb dsDNA was added to see DNA-assisted protein interaction. For the western blotting and coimmunoprecipitation studies, different derivatives of pVHS559 and pRAD plasmids expressing C-18 tag (18DrRecA and 18PprA) and His-tag (HisRecA and HisPprA) fusion proteins were co-transformed in different combinations into *D. radiodurans*. The recombinant cells co-expressing these proteins were induced with 0.5 mM IPTG, and harvested cell washed with 70% ethanol followed by lysed in the buffer (50 mM Tris base, 150 mM NaCl, 5 mM EDTA) containing 0.5% Triton X-100, 1 mM PMSF, 1 mM dithiothreitol (DTT), supplemented with 0.5 mg/ml lysozyme, and 50 μg of a protease inhibitor cocktail tablet followed by sonication. The clear cell-free extracts (CFE) were obtained by centrifugation at 12000 × *g* for 30 min. CFE used for immunoprecipitation using polyclonal antibodies against either T18 or Anti-His tag antibody (Ab) and precipitated immunoprecipitates were separated on a 10% SDS-PAGE gel, blotted onto a polyvinylidene difluoride (PVDF) membrane, and hybridized with monoclonal antibodies against the either T18 or Anti-His tag antibody (Ab) as required. Hybridization signals were detected using anti-mouse secondary antibodies conjugated with alkaline phosphatase using BCIP/NBT (5-bromo-4-chloro-3-indolylphosphate/nitroblue tetrazolium) substrates (Roche Biochemical, Mannheim, Germany).

### The Measure of Cell Survival, Recombination Frequency, Genomic Stability, and Transformation Efficiency

Wild type and its mutants (*recA*^–^ and *pprA*^–^) were treated with different doses of γ-radiation as described previously (Rajpurohit et al., 2008). In brief, mutant and wild-type *D. radiodurans* cells were grown in TGY medium to the late log phase at 32°C. The cells were suspended in sterile phosphate-buffered saline (PBS) and exposed to different doses of γ-radiation (GC500; ^60^CO; Board of Radiation and Isotopes Technology, Department of Atomic Energy, India). Appropriate dilutions were plated on TGY agar plates and incubated at 32°C. The numbers of CFU were recorded after 48 h of incubation at 32°C.

For recombination frequency estimation *pNOKpqq* plasmid was used (Rajpurohit et al., 2008). This suicidal vector-only survives when integrated at *the pqq* locus of the chromosomal site of host *D. radiodurans* cells. Recombination frequency was estimated for normal growth and γ-irradiated cells as discussed earlier (Vierling et al., 2000). Briefly, 10^6^ *D. radiodurans* R1 cells were mixed with 5 μg *pNOKpqq* plasmid, incubated on ice for 20 min and at 32°C for 50 min followed by dilution in 5 ml TGY medium overnight. Appropriate serial dilutions were plated on TGY plates with or without Kanamycin (8 μg/ml) and incubated at 32°C for 72 h to count colony-forming units (CFU). The recombination ability was calculated by the following formula: recombination efficiency (%) = (CFU with Kam/CFU without Kam) × 100, Here, Kam stands for kanamycin antibiotic. Genomic stability assayed as described earlier (Sheng et al., 2005). In brief, *nptII* gene stability was examined by genomic PCR using Npt-F and Npt-R primers (Supplementary Table 1). Homozygous cells incubated at 32°C in TGY medium till stationary phase and subcultured to fresh TGY medium. each subculture considered as a new generation. For each generation, approximately one thousand clones from every sample plated on TGY plates with and without antibiotics and plates incubated at 32°C. The formula applied for genetic stability (%) calculation [Genetic stability (%) = (CFU with Kam/CFU without Kam) × 100]. Plasmid pVHS559 was used to evaluate the transformation efficiency of γ-irradiated and unirradiated wild-type and *pprA* mutant cells. Transformants were selected on an appropriate antibiotic. 10 OD (A_600_) cells resuspended in fresh TGY medium and irradiated for a 6kGy dose. Unirradiated sham controls were plated parallelly. 30 mM CaCl_2_ is used to assist transformation. Appropriate dilution plated and transformation efficiently calculated by calculating CFU/μg plasmid DNA used.

### DNA Binding Assay

DNA binding activity of DrRecA and PprA protein was checked using electrophoretic gel mobility shift assay (EMSA) as described earlier (Rajpurohit et al., 2016). In brief, 40 nucleotides long random sequence oligonucleotide (Oligo40-F, Supplementary Table 1) was used as ssDNA substrate and dsDNA substrate was made by annealing it with its complementary strand (Oligo40-R, Supplementary Table 1). Both ssDNA and dsDNA were labeled with [^32^P]-γ-ATP using polynucleotide kinase and purified by G-25 column. The 0.2 pmole of the labeled probe (ssDNA and dsDNA) was incubated with increasing concentrations of DrRecA (0.5–2 μg) in 10 μl of reaction mixture containing 10 mM Tris-HCl, pH 7.5, 50 mM NaCl and 1 mM DTT for 10 min at 37°C. 2 μg PprA protein used with DrRecA or alone. Products were analyzed on a 12.5% native polyacrylamide gel, dried and signals were recorded by autoradiography.

### DNA Strand Exchange Reaction

Long homology-dependent RecA-dependent DNA strand exchange was carried out using circular M13mp18 ssDNA and linear dsDNA as described earlier (Kim and Cox, 2002). Reaction carried out in buffer (25 mM Tris-acetate, 1 mM DTT, 5% glycerol, 3 mM potassium glutamate, 10 mM magnesium acetate, and an ATP-regenerating system (10 units/ml of pyruvate kinase/3.3 mM phosphoenolpyruvate or 10 units/ml creatine kinase/12 mM phosphocreatine). 2.5 μM *E. coli* SSB (NEB), ATP, DrRecA, and PprA protein concentrations are indicated for each experiment. The reaction began with a pre-incubation of 6 μM ssDNA^nt^ with DrRecA protein at 37°C for 10 min. followed by the addition of ATP and SSB protein. After incubation of 10-min, linear 5 μM dsDNA^nt^ was added to start the DNA strand exchange reactions. PprA protein was added before and after the addition of dsDNA (when required). The reactions were stopped by the addition of 5 μl of gel loading buffer (0.125% bromophenol blue/25 mM EDTA/25% glycerol/5% SDS) and samples were electrophoresed in a 0.8% agarose gel with TAE buffer. Gel stained with ethidium bromide and photographed in Gel doc system (Syngene).

For the oligo-based DNA strand exchange reaction, firstly, 1 μl of 0.1 μM concentration Oligo40-F was labeled at 5′ end using polynucleotide kinase enzyme (PNK, NEB) using reaction buffer (70 mM Tris–HCl, pH 7.6, 10 mM MgCl_2_, and 5 mM DTT) and 1 μM [^32^P]-γ-ATP for 1 hr. Unused [^32^P]-γ-ATP removed by passing reaction mixture from G-25 column. To obtain dsDNA equal molar concentration of [^32^P]-labeled Oligo40-F and its complementary oligo Oligo40-R mixed in 50 μl reaction volume supplemented with 1X buffer (10 mM Tris–HCl, pH 7.6, 50 mM NaCl, and 1 mM EDTA) (Supplementary Table 1). Reaction sample heated for 5 min at 95°C and allowed for slow cooling to room temperature for annealing purpose. To perform the assay, indicated concentration of DrRecA incubated with oligo167-mer (2.5 μM nucleotides, Supplementary Table 1) in 10 μl of buffer (25 mM Tris–HCl, pH 7.5, 1 mM DTT, 2.5 mM MgCl_2_, 0.25 mM KCl) containing 1 mM ATP for 5 min., after this ^32^P-labeled oligo40-mer dsDNA oligonucleotide (2.5 μM nucleotides, Supplementary Table 1) added. PprA protein was added as and when required with indicated concentration. At the indicated times, a 2.5 μl aliquot was removed and mixed with an equal volume of 1% SDS containing proteinase K (1 mg/ml) and incubated at 37°C for 20 min. The samples were analyzed on 10% PAGE, dried gel exposed to x-ray, and autoradiogram developed.

### ATPase Assay

[α-^32^P] ATP (Board of Radiation and Isotope Technology, Dept. of Atomic Energy, India) was used for TLC, and the release of [α-^32^P]ADP was measured as described earlier (Modi et al., 2014). In brief, purified recombinant DrRecA (0.2 μM) was incubated in the buffer (25 mM Tris–HCl, pH 7.5, 1 mM DTT, 2.5 mM MgCl_2_, 1 mM ATP, 25 mM KCl, 2 nM ssDNA^nt^) added with 30 nM of [α^–32^P] ATP. DrRecA incubated with increasing concentration of PprA to check PprA effect on ATPase of DrRecA. Reaction mixtures were incubated at 37°C for 10 min. The reaction was stopped by the addition of 10 mM EDTA. Further, 1 μl of the reaction mixture was spotted on polyethyleneimine (PEI)-cellulose TLC sheets. Spots were air-dried, components were separated on a solid support in a buffer system in 0.75 M KH_2_PO_4_/H_3_PO_4_ (pH 3.5), and an autoradiogram was developed.

### ATP Binding Assay

ATP binding assay to DrRecA performed as described earlier (Rohn et al., 1999). In brief, 100 nM of fluorescent Mant-ATP (sigma) and 0.2 μM DrRecA was added in an assay buffer (20 mM Tris–HCl, 50 mM KCl, 1 mM DTT and 5 mM MgCl_2_) in a cuvette (final volume 0.4 ml). The samples were then analyzed on FLS980 Spectrometer, Edinburg Instruments, United Kingdom at room temperature using an excitation wavelength of 355 nm and recording the emission spectra from 400 to 500 nm. The baseline buffer spectrum was subtracted from all spectra shown. To check the PprA protein effect, PprA protein added in reaction with increasing concentration as indicated. ATP (5 mM) was added for the competition assays.

### Site-Directed Mutagenesis

PprA protein mutants were generated using a site-directed mutagenesis kit (New England Biolabs) following the kit manufacturer’s protocols. Details of primers used for site-directed mutagenesis are given in Supplementary Table 1.

### Evaluation of Transformation Efficiency

For the evaluation of transformation efficiency, 5 μg pVHSM plasmid was used to transform the wild-type and *pprA*^–^ mutant cells. Transformants were selected on spectinomycin antibiotic (100 μg/ml). For the gamma-irradiated cells, 10 OD (A_600_) cells were resuspended in a fresh TGY medium and irradiated for a 6kGy dose. Unirradiated sham controls were plated parallelly. 30 mM CaCl_2_ is used to assist transformation. Appropriate dilution plated and transformation efficiently calculated by calculating CFU/μg plasmid DNA used.

## Data Availability Statement

The original contributions presented in the study are included in the article/Supplementary Material, further inquiries can be directed to the corresponding author/s.

## Author Contributions

YR: hypothesized, design of experiments, execution, and manuscript writing and editing. DS: design and execution of experiments. HM: manuscript writing, editing, and discussion. All authors contributed to the article and approved the submitted version.

## Conflict of Interest

The authors declare that the research was conducted in the absence of any commercial or financial relationships that could be construed as a potential conflict of interest.
