# Supplementary material for: PprA Protein Inhibits DNA Strand Exchange and ATP Hydrolysis of Deinococcus RecA and Regulates the Recombination in Gamma-Irradiated Cells
Source: Front Cell Dev Biol. 2021 Apr 20;9:636178. doi: 10.3389/fcell.2021.636178 (PMC8093518; doi:10.3389/fcell.2021.636178)
Supplement: Supplementary file 1 [file Table_1.DOCX]

**Fig. S1** Western blot analysis of different tagged proteins. DrRecA and PprA protein tagged with T18 or His-tag and cloned in *D. radiodurans* shuttle vector (pVHS559 and pRAD plasmid). These constructs transformed to *D. radiodurans* and the expression of protein checked by either anti-T18 antibody or by anti-His antibody as appropriate. Western confirmation of (A)T18-tagged DrRecA, (B) His-tagged DrRecA, (C) T18-tagged PprA, and (D) His-tagged PprA.


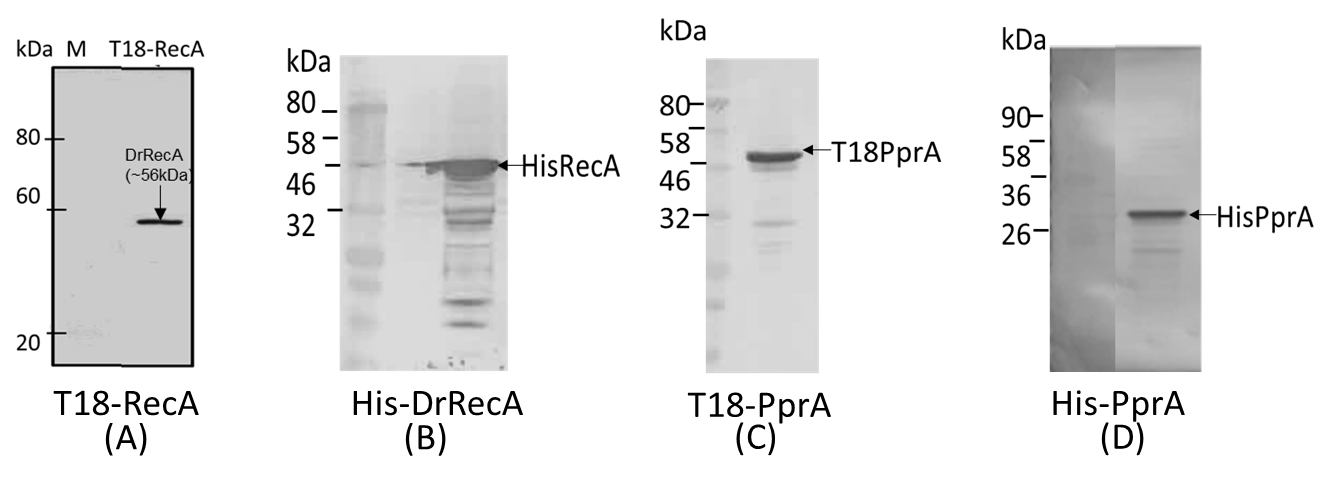


**Fig. S2** Assay of DrRecA and PprA protein interaction *in solution*. The interaction of DrRecA and PprA monitored by glutaraldehyde assisted crosslink assay and complex resolved on SDS-PAGE. Assay carried out using DrRecA and either wild type (PprA^wt^) or its oligomer mutant (PprA^R212A^) and DNA binding mutant (PprA^R166A^). Lane M- SDS marker, lane A- DrRecA protein, lane B- PprA protein, lane C- DrRecA and PprA protein treated with 0.5% glutaraldehyde, lane D- DrRecA and PprA protein mixed with 5 µg PCR dsDNA followed by addition of 0.5% glutaraldehyde and lane E- DrRecA and PprA protein mixed with 5µg PCR dsDNA followed by addition of 0.5% glutaraldehyde followed by DNAase treatment. The protein-protein interaction cross-linked product is shown by a red arrow.


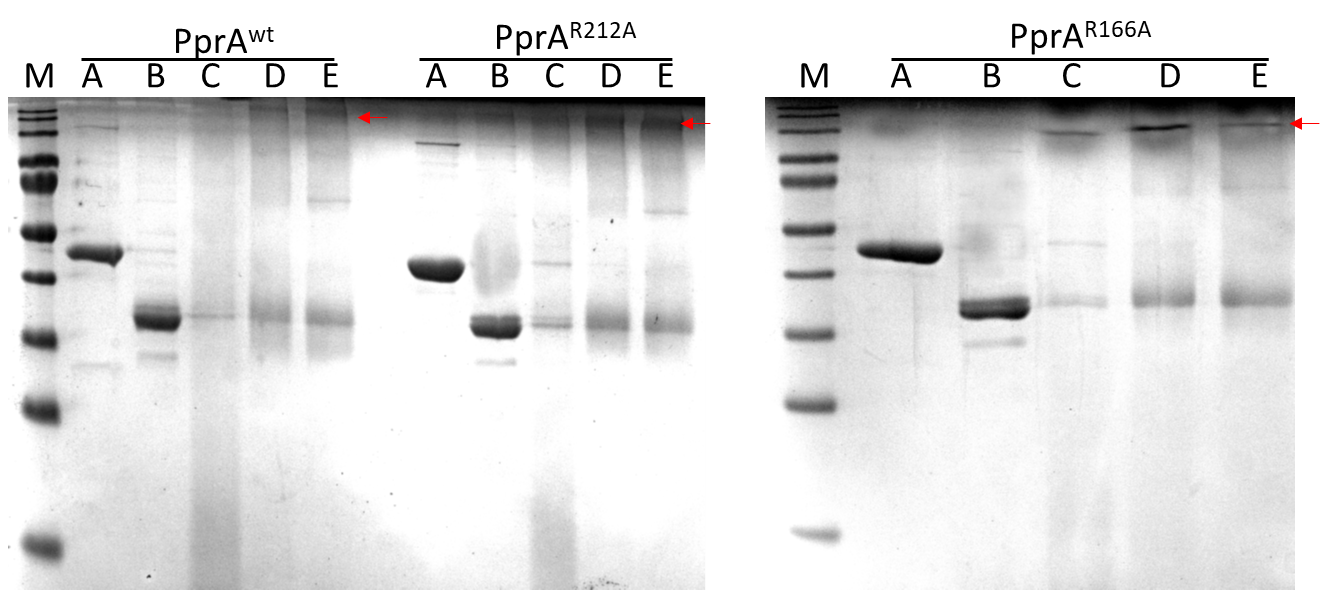


**Fig. S3** (A) Functional complementation of PprA loss in *pprA^-^* mutant and gamma survival of *recA^-^* mutant of *D. radiodurans.* Wild type and mutants were irradiated to different doses of γ-radiation and cell survival was monitored at different doses. Error bars, S.E. (standard error).

**Fig. S4** Electrophoretic gel shift assay (EMSA) showing DNA binding of DrRecA and PprA protein. EMSA assay was carried out to see the DNA binding properties of DrRecA and PprA protein. (A) increasing concentration of DrRecA (0.5 to 2 µg) incubated with 40mer ssDNA with the fixed concentration of PprA protein (2 µg) or in the absence of PprA protein. (B) increasing concentration of DrRecA (0.5 to 2 µg) incubated with 40mer dsDNA with the fixed concentration of PprA protein (2 µg) or in the absence of PprA protein. Data showed that PpRA protein did not bind with ssDNA, while form a distinct EMSA band when incubated with dsDNA. DrRecA protein could able to bind with both types of DNA.


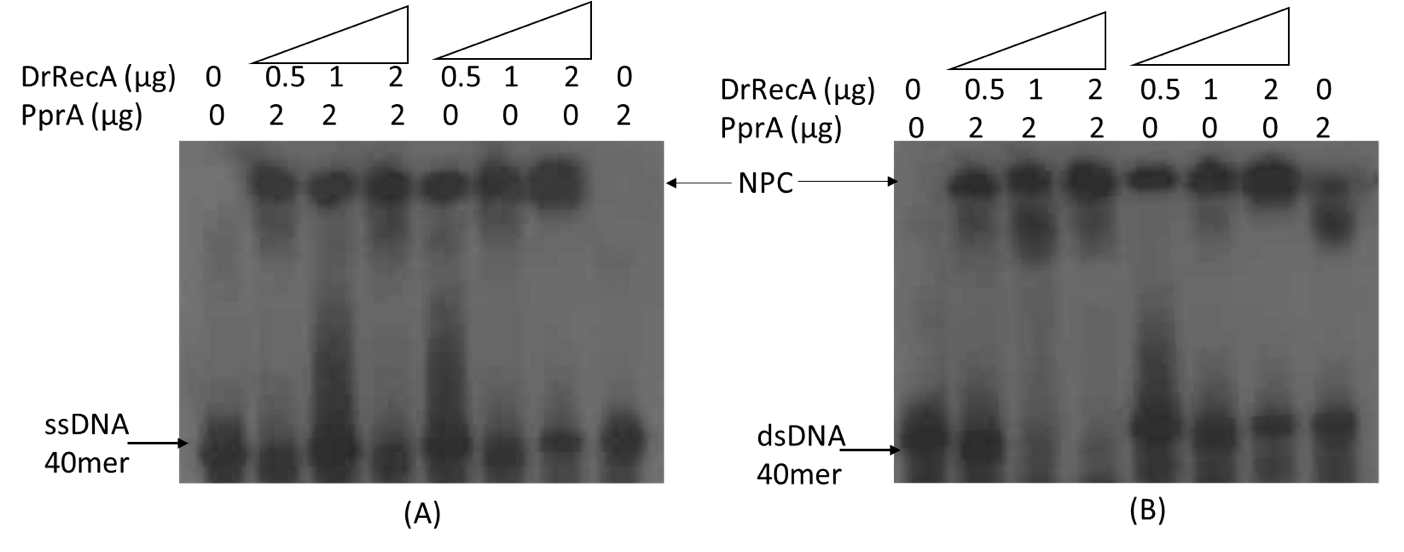


**Fig. S5** Effect of ATP on DrRecA catalysed DNA strand exchange. Increasing concentration of ATP (1 to 5 mM) was added in oligo-based DNA strand exchange reaction. Typically, oligo-based DNA strand exchange can be catalysed by DrRecA even in the absence of ATP. However, supplementing ATP till 5mM concentration did not hamper the DNA strand exchange catalysis.


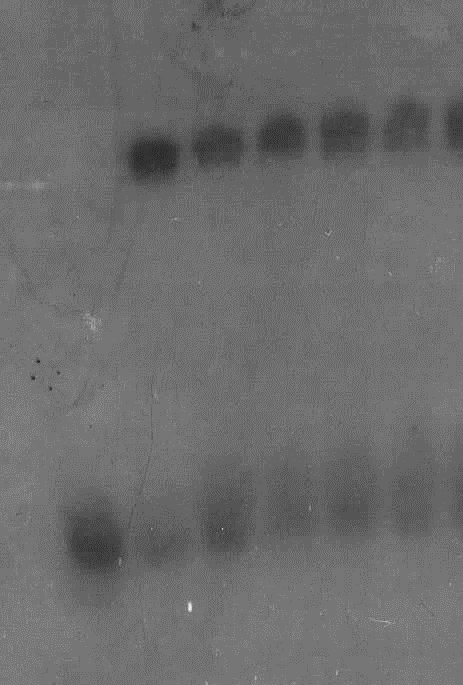


RecA (0.2µM) ̶ + + + +

ATP (mM) ̶ ̶ 1 2 5

Probe

(2nM)
