# Supplementary material for: PprA Protein Inhibits DNA Strand Exchange and ATP Hydrolysis of Deinococcus RecA and Regulates the Recombination in Gamma-Irradiated Cells
Source: Front Cell Dev Biol. 2021 Apr 20;9:636178. doi: 10.3389/fcell.2021.636178 (PMC8093518; doi:10.3389/fcell.2021.636178)
Supplement: Supplementary file 2 [file Table_2.DOCX]

Table S1. **Bacterial strains, plasmids, primers and used in this study**

| **Bacterial strains** | **Genotype** | **Source** | |  |
| --- | --- | --- | --- | --- |
| *D. radiodurans* R1 | Wild type strain ATCC13939 | Lab stock | |  |
| *E. coli* MG1655 | F- lambda- *ilvG*- *rfb*-50 *rph*-1 | Lab stock | |  |
| *E. coli* Novablue | *end*A1 *hsd*R17*(r _K12_ ^−^ m _K12_ ^+^) sup*E44 *thi-1 rec*A1 *gyr*A96 *rel*A1 *lac*F’'*[pro*A^+^B*^+^ lac*I^q^ *Z∆*M15*::*T*n*10] (Tet^R^) | Merck | |  |
| *E. coli* BTH 101 | F^-^, *cya-*99*, ara*D139*, gal*E15*, gal*K16*, rps*L1 (Str ^r^)*, hsd*R2*, mcr*A1*, mcr*B1 | 6 | |  |
| *E. coli* BL21(DE3) | *fhu*A2 *(lon)omp*T *gal*(λDE3)*(dcm) ∆hs*dS | Lab stock | |  |
| **Plasmids** | | | | |
| **Names** | **Characteristics** | **Source** | **Molecular mass of**  **protein** |  |
| pUT18 | pUC19 derivative, MCS at N-terminal of T18 fragments of adenylate cyclase, ~3 kb, AmpR | 7 | 18 kDa |  |
| pKNT25 | pSU40 derivative, MCS at N-terminal of T25 fragment of adenylate cyclase, ~3.4 kb, KanR | 7 | 25 kDa |  |
| pUTDr*recA* | pUT18 carrying dr*recA* at *Bam*HI and *Kpn*I | This study | 56 kDa |  |
| pKNDr*recA* | pKNT25 carrying dr*recA* at *Bam*HI and *Kpn*I | This study | 63 kDa |  |
| pUT*pprA* | pUT18 carrying *drpprA at Bam*HI *and Kpn*I | 1 | 50 kDa |  |
| pVHS559 | A shuttle vector between *D. radiodurans* and *E. coli* (Spec^R^) | 3 | - |  |
| pRADgro | pRAD1 carrying 26-bp *Bgl*II-*Xba*I fragment of promoter (Pgro) from *D. radiodurans* | 3 | - |  |
| pNOKpqq | A suicidal vector construct for *pqq* gene | 6 |  |  |
| pETdr*recA* | pET28a (+) carrying dr*recA* at *Bam*HI and *Xho*I | 4 | 38 kDa |  |
| pETdr*pprA* | pET28a (+) carrying dr*pprA* at *Nde*I and *Bam*HI | 5 | 32 kDa |  |
| pRadHis*recA* | pRADgro carrying coding sequence of (his)6-*recA* from pETrecA at *Apa*I and *Xba*I | This study | 41 kDa |  |
| pVH*recA*18 | pVHS559 carrying *recA*-C18 from pUTDrRecA at *Nde*I & *Xho*I | This study | 56 kDa |  |
| pRadHis*pprA* | pRADgro carrying coding sequence of (his)6-*pprA* from pETdr*pprA* at *Apa*I and *Xba*I | This study | 32 kDa |  |
| pVH*pprA*18 | pVHS559 carrying *pprA*-C18 from pUT*pprA* at *Nde*I & *Xho*I | This study | 50 kDa |  |
| pET*pprA*R212A | pET28a (+) carrying *pprA*R212A at *Nde*I and *Bam*HI | This study | 32 kDa |  |
| pET*pprA*R208A | pET28a (+) carrying *pprA*R208A at *Nde*I and *Bam*HI | This study | 32 kDa |  |
| pET*pprA*RK149R | pET28a (+) carrying *pprA*K149R at *Nde*I and *Bam*HI | This study | 32 kDa |  |
| pET*pprA*R166A | pET28a (+) carrying *pprA*R166A at *Nde*I and *Bam*HI | This study | 32 kDa |  |
| pVHpprA^GFP^ | pVHS559 carrying *pprA-gfp* | 2 | - |  |
| pDSrecA^RFP^ | pDSred plasmid carrying *recA* at KpnI and BamHI sites | This study | - |  |
| pRADrecA^RFP^ | pRAD plasmid carrying recA-rfp fusion gene at ApaI and EcoRV sites | This study | - |  |
| **Primer Name** | **Primer sequence (5’ to 3’)** | **Purpose / Plasmid** | |  |
| Oligo167 | CTGCTTTATCAAGATAATTTTTCGACTCATCAGAAATATCCGTTTCCTATATTTATTCCTATTATGTTTTATTCATTTACTTATTCTTTATGTTCATTTTTTATATCCTTTACTTTATTTTCTCTGTTTATTCATTTACTTATTTTGTATTATCCTTATCTTATTTA | DNA strand exchange | |  |
| Oligo40-F | TAATACAAAATAAGTAAATGAATAAACAGAGAAAATAAAG | DNA strand exchange | |  |
| Oligo40-R | CTTTATTTTCTCTGTTTATTCATTTACTTATTTTGTATTA | DNA strand exchange | |  |
| Npt-F | ATGGTGGCATTTCTCCGTGGC | Genomic PCR | |  |
| Npt-R | TCATGCGTGACTTACCAATGGA | Genomic PCR | |  |
| *pprA*-F | ACGCTCGAGATGGCAAGGGCTAAAGCAAAAG | pET*pprA* | |  |
| *pprA*-R | TTAGAATTCTCAGCTCTCGCGCAGGCCGTGCC | pET*pprA* | |  |
| R212A-F | CGCGCCTTCCAGGCTGAGCTGATGCG | pET*pprA*R212A / SDM | |  |
| R212A-R | CGCATCAGCTCAGCCTGGAAGGCGCG | pET*pprA*R212A / SDM | |  |
| K149R-F | GTTGGCGCAGCTCGCGGTGCTGATCGAG | pET*pprA*K149A / SDM | |  |
| K149R-R | CTCGATCAGCACCGCGAGCTGCGCCAAC | pET*pprA*K149A / SDM | |  |
| R166A-F | CTGGTCGGCGGGGGCCGGCGAAACCTTTC | pET*pprA*R166RA / SDM | |  |
| R166A-R | GAAAGGTTTCGCCGGCCCCCGCCGACCAG | pET*pprA*R166RA / SDM | |  |
| R208A-F | CAGCATCAAGGACGCCGCCTTCCAGCG | pET*pprA*R208A / SDM | |  |
| R208A-R | CGCTGGAAGGCGGCGTCCTTGATGCTG | pET*pprA*R208A / SDM | |  |
| RFP_dsred-F | GCGGATCCGATGAGCAAGGACGCCAC | pDSrecA^RFP^ | |  |
| RFP_dsred-R | GCTCTAGACGCTTCGGCGGCTTC | pDSrecA^RFP^ | |  |
| pRADrecA^RFP^-F | CGGAGCTCGGGCCCCACCACCACCACCACCATGCCTGCAGGTCG | pRADrecA^RFP^ | |  |
| pRADrecA^RFP^-R | CGGATATCTAGACTCGAGGCCGCTACAG | pRADrecA^RFP^ | |  |
| BTH*recA*-F | GCGGATCCGATGAGCAAGGACGCCAC | BTH*recA* | |  |
| BTH*recA*-R | GCGGTACCCGCGCTTCGGCGGCTTC | BTH*recA*  BTH*pprA* | |  |
| BTH*pprA*-F | GCGGATCCGGTGCTACCCCTGGCC |  |  |  |
| BTH*pprA*-R | GCGGTACCGCTCTCGCGCAGGCC | BTH*pprA*  pRADHis*pprA* | |  |
| pETHisFw | AAAGTACTGGGCCCATGGGCAGCAGCCAT |  |  |  |
| pETHisRw | GCCTTAAGTCTAGATATCTCAGTGGTGGTG | pRADHis*pprA* | |  |
|  |  |  |  |  |

**References:**

1. Kota, S, et al. (2014) PprA contributes to Deinococcus radiodurans resistance to nalidixic acid, genome maintenance after DNA damage and interacts with deinococcal topoisomerases. PLoS One 9.1: e85288.
2. Kota, S., et al. (2014) a pleiotropic protein for radioresistance, works through DNA gyrase and shows cellular dynamics during postirradiation recovery in Deinococcus radiodurans. J Genet 93, 349–354.
3. Maurya, G. K., Modi, K., Banerjee, M., Chaudhary, R., Rajpurohit, Y. S., & Misra, H. S. J. M. (2018). Phosphorylation of FtsZ and FtsA by a DNA damage-responsive Ser/Thr protein kinase affects their functional interactions in Deinococcus radiodurans. *3*(4).
4. Rajpurohit, Y. S., Bihani, S. C., Waldor, M. K., & Misra, H. S. J. J. o. B. C. (2016). Phosphorylation of Deinococcus radiodurans RecA regulates its activity and may contribute to radioresistance. *291*(32), 16672-16685.
5. Rajpurohit, Y. S., Misra, H. S. J. T. I. J. o. B., & Biology, C. (2013). Structure-function study of deinococcal serine/threonine protein kinase implicates its kinase activity and DNA repair protein phosphorylation roles in radioresistance of Deinococcus radiodurans. *45*(11), 2541-2552.
6. Rajpurohit YS, Gopalakrishnan R, Misra HS. Involvement of a protein kinase activity inducer in DNA double strand break repair and radioresistance of Deinococcus radiodurans. J Bacteriol. 2008 Jun;190(11):3948-54.
7. Saalbach G, Hempel AM, Vigouroux M, Flärdh K, Buttner MJ, Naldrett, M. 2013. Determination of phosphorylation sites in the DivIVA cytoskeletal protein of *Streptomyces coelicolor* by targeted LC–MS/MS. J. Proteome Res. 12:4187–4192.
